# Interactions between rheumatoid arthritis antibodies are associated with the response to anti-tumor necrosis factor therapy

**DOI:** 10.1186/s12891-021-04248-y

**Published:** 2021-04-21

**Authors:** Antonio Julià, María López-Lasanta, Francisco Blanco, Antonio Gómez, Isabel Haro, Antonio Juan Mas, Alba Erra, Ma Luz García Vivar, Jordi Monfort, Simón Sánchez-Fernández, Isidoro González, Mercedes Alperi, Raúl Castellanos-Moreira, Antonio Fernández-Nebro, César Díaz-Torné, Núria Palau, Raquel Lastra, Jordi Lladós, Raimon Sanmartí, Sara Marsal

**Affiliations:** 1 Rheumatology Research Group, Vall d’Hebron University Hospital Research Institute, 08035 Barcelona, Spain; 2 Rheumatology Department, INIBIC-Hospital Universitario A Coruña, A Coruña, Spain; 3 Unitat de Síntesi i Aplicacions Biomèdiques de Pèptids, IQAC-CSIC, Barcelona, Spain; 4 Rheumatology Department, Hospital Universitario Son Llàtzer, Mallorca, Spain; 5 Rheumatology Department, Hospital Universitario Basurto, Bilbao, Spain; 6 Rheumatology Department, Hospital del Mar, Barcelona, Spain; 7 Rheumatology Department, Hospital General La Mancha Centro, Ciudad Real, Spain; 8 Rheumatology Department, Hospital Universitario La Princesa, Madrid, Spain; 9 Rheumatology Department, Hospital Universitario Central de Asturias, Oviedo, Spain; 10 Rheumatology Department, Fundació Clínic Recerca Biomèdica, Barcelona, Spain; 11 Rheumatology Department, Hospital Regional Universitario de Málaga, Málaga, Spain; 12 Rheumatology Department, Hospital de la Santa Creu i Sant Pau, Barcelona, Spain

**Keywords:** Rheumatoid arthritis, Treatment response, Anti-TNF therapy, Autoantibodies

## Abstract

**Background:**

Blocking of the Tumor Necrosis Factor (TNF) activity is a successful therapeutic approach for 50–60% of rheumatoid arthritis (RA) patients. However, there are yet no biomarkers to stratify patients for anti-TNF therapy. Rheumatoid factor (RF) and anti-cyclic-citrullinated antibodies (anti-CCP) have been evaluated as biomarkers of response but the results have shown limited consistency. Anti-carbamylated protein (anti-CarP) and anti-peptidylarginine deiminase type 4 (anti-PAD4) antibodies have been much less studied. Despite being linked to common immune processes, the interaction between these markers has not been evaluated yet. Our aim was to analyze the interaction between these four antibodies in relation to the response to anti-TNF therapy.

**Methods:**

For this objective, a prospective cohort of *n* = 80 RA patients starting anti-TNF therapy was recruited. Serum determinations at baseline were performed for RF, anti-CCP, anti-CarP and anti-PAD4 antibodies using enzyme-linked immunosorbent assays (ELISA). The clinical response to anti-TNF therapy was determined at week 12 using the change in DAS28 score. Association was performed using multivariate linear regression adjusting for baseline DAS28, sex and age.

**Results:**

The interaction between pairs of antibodies was tested by the addition of an interaction term. We found two highly significant antibody interactions associated with treatment response: anti-CarP with anti-PAD4 (*p* = 0.0062), and anti-CCP with RF (*p* = 0.00068). The latter antibody interaction was replicated in an independent retrospective cohort of RA patients (*n* = 199, *p* = 0.04).

**Conclusions:**

The results of this study suggest that antibody interaction effects are important factors in the response to anti-TNF therapy in RA.

**Supplementary Information:**

The online version contains supplementary material available at 10.1186/s12891-021-04248-y.

## Background

Rheumatoid arthritis (RA) is the most common chronic inflammatory arthritis, with a Worldwide prevalence of 0.5–1%. Tumor necrosis factor (TNF) is a proinflammatory cytokine that is central to the inflammatory process of RA. Systemic blocking of this cytokine has proven to be a highly efficacious approach to control the disease activity [1]. Despite this major therapeutic advance, up to 40% of RA patients treated with a TNF blocking agent don’t show a significant clinical improvement. Very little knowledge exists on the factors that determine this unfavorable response, and biomarkers have yet to be identified.

RA is characterized by the expression of antibodies against self-antigens, and consequently they have been among the first biomarkers to be evaluated for association with treatment response. Antibodies against the Fc portion of immunoglobulin G –rheumatoid factor (RF)- and against cyclic citrullinated peptides (anti-CCP) are currently the two most relevant diagnostic tests for RA [2]. Both autoantibodies have been clearly associated to unfavorable prognosis [3]. However, their association to the response to anti-TNF therapy is much less clear. Previous studies have shown inconclusive or conflicting results [4–6]. Consequently, interest has shifted in analyzing more recently discovered antibodies as potential biomarkers for treatment response.

Anti-peptidylarginine deiminase type 4 (anti-PAD4) antibodies [7] and anti-carbamylated protein (anti-CarP) antibodies [8] are recent markers in RA. Anti-PAD4 antibodies, although not specific for RA, have been associated to a more severe disease phenotype [9]. There is yet scarce data on the association of anti-PAD4 in anti-TNF response. A first small study on 40 patients found that patients positive for this antibody had a worse response to therapy [10]. A more recent study involving triple DMARD and anti-TNF therapy treated patients, suggested that anti-PAD4 positive patients had instead a more favorable response [11]. However, the association was not tested individually for each drug arm, so the specific association to TNF blocking is unclear. Anti-CarP antibodies occur in up to 40% of RA patients and, like anti-CCPs, they can appear several years before the onset of the disease. To date, anti-CarP antibodies have not been tested for association with the response to anti-TNF therapy in RA.

The presence of previous conflicting results could be an indication that a more complex relationship exists between antibodies and the response to anti-TNF therapy in RA. From a biological perspective, anti-CarP, anti-PAD4, RF and anti-CCP target proteins involved in closely related biological processes. The simultaneous expression of two or more of these antibodies could therefore represent a higher load of specific pathogenic mechanisms. Recent experimental evidence supports the presence of this type of pathogenic interactions between RA autoantibodies [12]. From a clinical perspective, the presence of synergic effects between antibodies could translate into stronger responses to therapy. In the present study we have addressed this question and analyzed, for the first time, the association of antibody interactions with the response to anti-TNF therapy.

## Methods

### Patients and samples

A prospective cohort of *n* = 80 RA patients was recruited from 11 university hospitals from Spain. Enrolled patients fulfilled the ACR/EULAR 2010 classification criteria for RA [2] and were starting an anti-TNF therapy. All patients had an active disease at baseline, described as a 28-joint Disease Activity Score (DAS28) ≥ 3.2. The same day of treatment initiation, blood samples were obtained and the plasma fraction separated and stored at − 80 °C until analysis.

The validation dataset consisted on a retrospective cohort of *n* = 199 RA patients that were recruited by the IMID Consortium, a network of rheumatology departments from *n* = 15 university hospitals in Spain [13]. All patients fulfilled the ACR/EULAR 2010 classification criteria for RA, and the primary response to anti-TNF therapy at week 12 was collected. Plasma samples were processed following the same procedure.

### Antibody measurements

The four antibodies were analyzed using enzyme-linked immunosorbent assays (ELISA). RF and anti-CCP were measured using the IBL International and the Euro Diagnostica anti-CCP2 ELISA kits, respectively. Positivity for anti-CCP and RF were defined according to the manufacturer’s protocols (≥ 18 U/ml and ≥ 25 U/ml for RF and anti-CCP, respectively). Anti-PAD4 titers were measured using the PAD4 autoantibody ELISA kit (Cayman Chemical). Anti-carbamylated proteins IgG autoantibodies (anti-CarP) were determined using a home-made ELISA test using as antigen carbamylated fetal calf serum (FCS). A non-carbamylated version of the FCS was used to control for homocitrulline specificity. Reactivity to non-modified FCS was subtracted from the reactivity to carbamylated FCS and a standard curve of serial dilutions of a pool of four positive samples was used to convert optical density values to arbitrary units (AU). Compared to IgM-RF and anti-CCP2, there is no established threshold for positivity for anti-PAD4 or anti-CarP biomarkers, and antibody titers were directly used to test for association with anti-TNF response.

In the validation cohort, anti-CCP was measured using an electrochemiluminescence assay (Cobas, Roche) and the RF was determined using an immunoturbidimetric method (Cobas, Roche). Positivity thresholds were defined using the manufacturer’s protocol (≥ 17 U/ml and ≥ 14 U/ml for anti-CCP and RF, respectively).

### Statistical analysis

The primary outcome measure was the change in the DAS28 score (ΔDAS28) between baseline and 12 weeks of anti-TNF therapy [14]. The association between anti-CarP, PAD4, RF and anti-CCP antibodies and treatment response was determined using multivariate linear regression. The multivariate model included sex, age and the baseline DAS28 measure, as described previously [4].

Interaction testing was performed by including an additional interaction term in the multivariate regression model. All pairwise interaction models between the four biomarkers were tested (*n* = 6). Multiple testing significance correction was performed using Bonferroni’s adjustment.

## Results

### Patient characteristics

Baseline characteristics of the prospective patient cohort are summarized in Table 1. Clinical measures are comparable to previous RA cohorts. The four autoantibody titers were determined in 100% of the patients. 65% of the RA patients were positive for RF and 72% of for anti-CCP. An average reduction of 1.96 (+/− 1.33) points in the DAS28 score was observed for the global cohort, which is consistent with previous studies [15].

**Table 1:** Baseline characteristics of the prospective RA patient cohort

Baseline variable	Total (***n*** = 80)	Responders (***n*** = 67)	Non-Responders (***n*** = 13)
Age, years (mean ± SD)	54.2 ± 11.93	53.12 ± 11.65	59.7 ± 12.32
Gender (Female,n %)	66 (82.5)	54 (80.6)	12 (92)
Previous csDMARDs (mean ± SD)	1.85 ± 1.28	1.82 ± 1.24	2 ± 1.53
Disease duration, years (median/IQR)	9.74 (9.25)	9.24 (9.5)	12.31 (11.41)
ESR, mm/h (median/IQR)	35.1 (28.75)	36.52 (34)	27.7 (21.17)
CRP, mg/dL (median/IQR)	1.63 (1.27)	1.63 (1.23)	1.62 (1.47)
MTX dosage (mean mg/week)	18.46	18.25	19.64
Prednisone use (n, %)	60 (75)	50 (74.6)	10 (77)
Smoking (n, %)			
Never	54 (67.5)	44 (65.7)	10 (77)
Past	10 (12.5)	6 (9)	3 (23)
Current	16 (20)	17 (25.3)	0 (0)
Adalimumab	16 (20)	15 (22.4)	1 (8)
Certolizumab	24 (30)	21 (31.3)	3 (23)
Etanercept	19 (23.75)	16 (23.4)	3 (23)
Golimumab	21 (26.25)	15 (22.4)	6 (46)

Clinical and epidemiological characteristics of the prospective cohort. Patients are shown globally and split according to the EULAR response at week 12 (Good and Moderate responders aggregated into a unique Responder group). *MTX* methotrexate; *csDMARDs* conventional synthetic DMARDs; *IQR* interquartile range; *SD* standard deviation

### Association of antibodies with anti-TNF response

Multivariate linear regression was used to simultaneously test for association between anti-CCP, RF, anti-CarP and anti-PAD4 antibodies and treatment response. At the single-marker level, none of the autoantibodies were found to be significantly associated with an improvement in the DAS28 (Supplementary Table S1). We next tested for the presence of interaction effects between all six pairwise antibody combinations in relation to treatment response. We found a highly significant antibody interaction between anti-CarP and anti-PAD4 (*P* = 0.0062), and between anti-CCP and RF (*P* = 0.00068) with anti-TNF response (Table 2). anti-CarP:anti-PAD4 interaction was associated with a worse response to therapy (interaction coefficient β < 0) and anti-CCP:RF interaction with a better response to TNF blocking (β > 0). The remaining four pairwise antibody interactions were not significant (*P* > 0.05). After adjusting for multiple testing, anti-CarP:anti-PAD4 and anti-CCP:RF interactions remained statistically significant (*P* = 0.0041 and *P* = 0.037, respectively).

**Table 2:** Association results for RA antibody interactions with anti-TNF response

	Regression coefficient (95%CI), ***P***-value		
Antibody pair	Interaction effect	Antibody #1 main effect	Antibody #2 main effect
#1: Anti-CCP #2: RF	**2.74 (1.20,4.26),** ***P*** **= 0.00068**	−1.21 (−2.14, −0.28), *P* = 0.012	−2.24 (−3.57, − 0.91), *P* = 0.0013
#1: RF #2: Anti-PAD4	− 2.7e-4 (−7.5e-4,1.9e-4), *P* = 0.25	0.17 (− 0.89,1.21), *P* = 0.75	1.9e-4 (− 2.1e-4,5.9e-4), *P* = 0.34
#1: Anti-PAD4 #2: Anti-CarP	**−1.3e-6 (− 2.20–6,3.8e-6),** ***P*** **= 0.0062**	2.2e-4 (− 3.6e-5,4.8e-4), *P* = 0.090	1.3e-3 (9.6e-5,2.5e-3), *P* = 0.035
#1: Anti-CCP #2: Anti-PAD4	−2.5e-4 (− 7.5e-4,2.5e-4), *P* = 0.33	0.12 (− 0.94,1.18), *P* = 0.83	1.8e-4 (− 2.6e-4,6.2e-4), *P* = 0.41
#1: Anti-CCP #2: Anti-CarP	8.9e-4 (−8.9e-4,2.7e-3), *P* = 0.32	−0.45 (− 1.38,0.48), *P* = 0.34	−8.4e-4 (− 2.5e-3,7.9e-4), *P* = 0.31
#1: RF #2: Anti-CarP	−5.39e-4 (− 3.1e-3,1.9e-3), *P* = 0.67	− 0.17 (− 0.99,0.65), *P* = 0.67	3.87e-3 (− 2e-3,2.8e-3), *P* = 0.75

Each row shows the association results for each of the six possible pairwise interactions between the four RA antibodies and anti-TNF treatment response, adjusting for baseline DAS28, sex and age. Regression coefficients (β value) with 95% confidence intervals (CI) and *P* values for association are shown for the interaction term (first column) and for the independent effect of each antibody (second and third columns). In bold, interaction *P*-values that are significant after correcting for multiple testing. A highly significant interaction was found for anti-CCP:RF and anti-PAD4:anti-CarP interactions with anti-TNF response. None of the remaining four antibody interactions showed a significant association, even at the nominal (*P* < 0.05) level

**Fig. 1:**
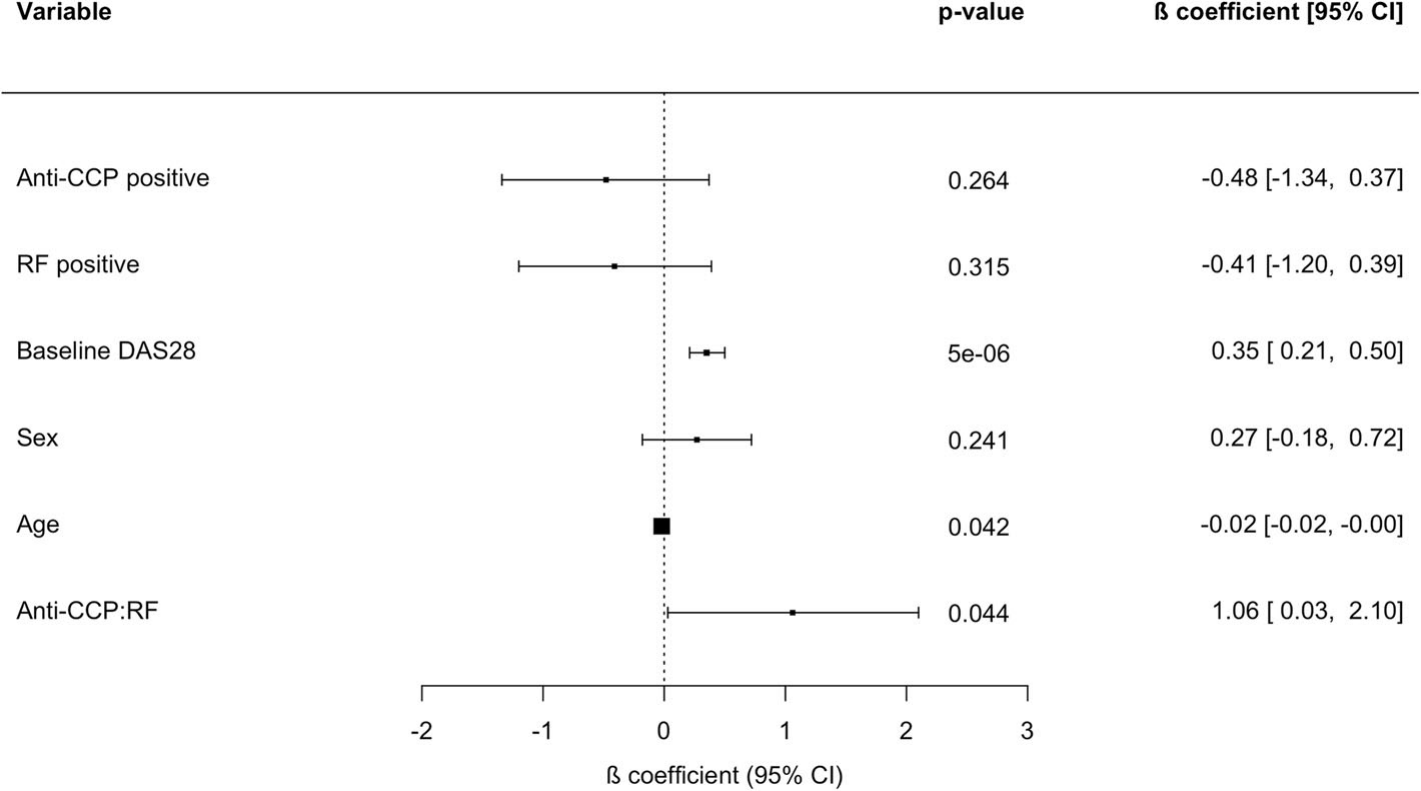
Validation study of the anti-CCP and RF interaction and anti-TNF response in RA. Forest plot showing the regression coefficients and 95% confidence intervals of the variables in the linear model testing the association of the two antibody combination with the response to anti-TNF therapy at week 12. Like in the prospective patient cohort, the interaction between anti-CCP and RF is statistically significant and positively associated with anti-TNF response (Beta:1.06 (0.03 to 2.10); *P* < 0.05). Anti-CCP:RF: regression coefficient capturing the interaction effect

Using an independent cohort of 199 RA patients, we replicated the interaction between anti-CCP and RF (*P* = 0.044, Fig. 1). Like in the prospective cohort, the interaction was also found to be positive, indicating that the simultaneous presence of both antibodies is associated with a better response to anti-TNF therapy.

## Discussion

The identification of factors associated with the response to anti-TNF therapy is a major objective for treatment personalization in RA. Diagnostic autoantibodies, like rheumatoid factor and anti-CCP, are appealing for this task since they are already integrated into the standard clinical routine. However, conflicting results have been reported and their association to treatment response to anti-TNF is yet not clear. We hypothesized that this inconsistency could be due to the presence of interaction effects between the autoantibodies. In the present study we have tested this hypothesis for the first time. Using a prospective cohort of RA patients starting anti-TNF therapy, we have found that the interaction between anti-CCP and RF and the interaction between anti-CarP and anti-PAD4 antibodies are both strongly associated with the clinical response at week 12. The present results suggest that interactions between antibodies are important in the response to anti-TNF therapy, and provide an explanation for the previous conflicting evidence.

Our study shows that the presence of both anti-CCP and RF antibodies is needed for a favorable response to anti-TNF therapy. Several previous studies have analyzed the association of either antibody in relation with the response to anti-TNF drugs. The results, however, have been largely inconsistent or inconclusive [16]. In those few studies where both antibodies were determined, the presence of interactions was not evaluated. Here we show that, when the interaction is considered, a strong and positive association between these two classic antibodies and the clinical response emerges. From a statistical perspective, when interaction effects are present and are strong, not taking them into account in the association model can lead to inconsistent findings [17]. Failing to take this into account could therefore explain the lack of reproducibility of previous studies with anti-CCP and RF and treatment response.

The interaction association identified between anti-CCP and RF with the response to anti-TNF therapy is in accordance with recent findings at the functional level. In a recent study, macrophages -the main producers of TNF in the RA joint- have been shown to secrete much higher TNF cytokine levels when stimulated with both anti-CCP and RF antibodies than when stimulated with anti-CCP alone [12]. According to these results, disease activity in RA patients that express both anti-CCP and RF might be partially due to the overexpression of TNF by macrophages reacting strongly to the combination of the two antibodies. Instead, in patients expressing only one of the antibodies or in seronegative patients, this synergic production of TNF by the synovial macrophage will not occur, and other inflammatory pathways will therefore have a more predominant role in disease activity. Our results show that, although still effective in some patients, anti-TNF therapy has a much less pronounced therapeutic effect in patients with only one antibody compared to patients positive for both anti-CCP and RF.

In our study we also found that the interaction between anti-CarP and anti-PAD4 is associated to anti-TNF response. In this case, we found that the higher the expression of both antibodies, the worse the patients responded to TNF blocking. Compared to anti-CCP and RF, these two antibodies do not physically interact at the molecular level. However, the two antibodies share a strong association to neutrophil activity. PAD4 is responsible for most of the citrullinated epitopes in RA [18] and is specifically expressed in neutrophils. Protein carbamylation is caused by an increase in tissue cyanate due to the activity of neutrophil myeloperoxidase during inflammation in RA. A higher abundance of both antibodies therefore suggests a more predominant role of neutrophils in RA pathology. According to our results, patients with a strong neutrophil-mediated inflammation are less sensitive to therapeutic TNF blocking. This is in line with recent experimental evidence showing that neutrophil activation and TNF have independent effects in RA pathology [19]. New therapies that directly affect neutrophil activation like, granulocyte-macrophage colony-stimulating factor blocking, are currently under way in RA [20]. Our results suggest that the simultaneous analysis of anti-PAD4 and anti-CarP antibodies could be a useful biomarker of response in this new therapeutic approach.

The present study has limitations. Despite most previous studies analyzing the association of antibodies to anti-TNF response have used similar or smaller sample sizes, the number of patients used in our prospective study is relatively modest. Having a larger patient cohort would have enabled a more precise estimation of the interaction effects, with narrower confidence intervals. To this regard, while the quantitative nature of the ΔDAS28 can help increase the power to identify biomarkers of drug efficacy, small improvements in DAS28 might not have a translation into clinically meaningful responses. More patient data on the four antibodies will help to better define this boundary. Finally, the comparison of the interaction association between different types of anti-TNF drugs could not be explored. There is evidence that TNF blocking agents work through biological mechanisms that are not entirely overlapping [21, 22]. An individual analysis of each drug type might reveal stronger interactions and better biomarker utility. For this aspect to be adequately tested, larger drug-specific prospective patient cohorts will need to be analyzed.

## Conclusion

In summary, in the present study we have found that RA antibodies show significant interaction effects with the response to anti-TNF therapy. The observed interactions are in line with pathogenic mechanisms recently described in RA. Our findings also provide an explanation for the lack of consistency observed in previous studies, in which antibodies were analyzed independently. These results leverage the potential of antibodies as biomarkers for anti-TNF response in RA.

## Supplementary Information

**Additional file 1: Supplementary Table S1.** Association results for the four antibodies and anti-TNF response at week 12 in the prospective cohort (independent effects).

## Data Availability

The datasets used and/or analysed during the current study available from the corresponding author on reasonable request.

## Abbreviations

TNF: Tumor Necrosis Factor
RA: Rheumatoid Arthritis
DAS28: Disease Activity Score for 28 joints
RF: Rheumatoid factor
anti-CCP: Anti-cyclic-citrullinated antibodies
anti-CarP: Anti-carbamylated protein antibodies
anti-PAD4: Anti-peptidylarginine deiminase type 4 antibodies
ELISA: Enzyme-linked immunosorbent assay
ACR: American College of Rheumatology
EULAR: European League Against Rheumatism
