# Supplementary material for: Interactions between rheumatoid arthritis antibodies are associated with the response to anti-tumor necrosis factor therapy
Source: BMC Musculoskelet Disord. 2021 Apr 21;22:372. doi: 10.1186/s12891-021-04248-y (PMC8061050; doi:10.1186/s12891-021-04248-y)
Supplement: Supplementary file 1 — Additional file 1: Supplementary Table S1. Association results for the four antibodies and anti-TNF response at week 12 in the prospective cohort (independent effects). [file 12891_2021_4248_MOESM1_ESM.pdf]

Title: **Interactions between rheumatoid arthritis antibodies are associated with the response to anti-Tumor Necrosis Factor therapy.**

**Supplementary Table S1. Association results for the four antibodies and anti-TNF response at week 12 in the prospective cohort (independent effects).**

| **Variable** | **Regression coefficient (95%CI)** | **P value** |
|---|---|---|
| Anti-CCP negative | Reference | - |
| Anti-CCP positive | -0.22 (-1.02 to 0.58) | 0.59 |
| RF negative | Reference | - |
| RF positive | -0.23 (-1.01 to 0.54) | 0.55 |
| Anti-CarP | -1.07e-4 (-8e-4 to 6e-4) | 0.77 |
| Anti-PAD4 | -5.77e-6 (-2.1e-4 to 2.1e4) | 0.96 |
| Baseline DAS28 | 0.42 (0.16 to 0.67) | 0.0015 |
| Sex: Female | Reference | - |
| Sex: Male | 0.19 (-0.60 to 0.99) | 0.62 |
| Age | -0.017 (-0.041 to 0.0061) | 0.14 |

Multivariate linear regression analysis for anti-cyclic citrullinated peptide (CCP), rheumatoid factor (RF), anti-carbamylated protein (CarP) and anti-peptidylarginine deiminase type 4 (PAD4) antibodies with anti-TNF response (i.e. change in DAS28 from week 0 to week 12). Adjustment was performed for baseline DAS28, sex and age. None of the four antibodies were associated independently with the anti-TNF response. As expected, the baseline DAS28 was significantly associated with the change in DAS28. DAS28: 28-joint disease activity score; Reference: reference group.
